# Supplementary material for: Myogenin is required for assembly of the transcription machinery on muscle genes during skeletal muscle differentiation
Source: PLoS One. 2021 Jan 19;16(1):e0245618. doi: 10.1371/journal.pone.0245618 (PMC7815108; doi:10.1371/journal.pone.0245618)
Supplement: S1 Table — (PDF) [file pone.0245618.s001.pdf]

Oligonucleotides used in study

Cloning primers:

Murine MyoD	F 5' ATGGAGCTTCTATCGCCG 3'	
Murine MyoD	F 5' ATGGAGCTTCTATCGCCG 3'	
Murine myog sgRNA Exon-1	Top 5' CACCGTCAGTCCGCTCATAGCCCGGGGG 3'	
	Bottom 5' AAACCCCCCGGGCTATGAGCGGACTGAC 3'	

Quantitative reverse transcriptase-PCR (qRT-PCR) primers:

18S rRNA	F 5' CGCCGCTAGAGGTGAAATTCT 3'
	R 5' CGAACCTCCGACTTTCGTTCT 3'
Hprt11	F 5' TGACACTGGCAAAACAATGCA 3'
	R 5' GGTCCTTTTCACCAGCAAGCT 3'
Murine Acta1	F 5' GGCACCCAGGGCCAGAGTCA 3'
	R 5' TCATCCCCGGCAAAGCCAGC 3'
Murine Lmod2	F 5' ACCTTATCCCGATTTGCTGAAG 3'
	R 5' ACCTTGAGCATGTCTGCAATG 3'
Murine Tnni2	F 5' GCCGCCGAGAATCTGAGA 3'
	R 5' GACATGGAGCCTGGGATGTG 3'
Murine Mylpf	F 5' GGCTGCCGGGGCAGGACTAT 3'
	R 5' CGGCCCATGGCTGCAAAGGT 3'
Murine MyoD	F 5' GCCGGTGTGCATTCCAA 3'
	R 5' CACTCCGGAACCCCAACAG 3'
Murine Myog	F 5' GACCTGATGGAGCTGTATGAG 3'
	R 5' CTGAAGGTGGACAGGAAGG 3'
Murine Myf5	F 5' AGCTTGCAAGAGGAAGTCCACTA 3'

	R 5' CTACGCTCGCGCATGGT 3'
Murine Myf6	F 5' CCCTGAAGCGTCGGACTGT 3'
	R 5' ATGGCACTCCGCAGAATCTC 3'
Murine Myomaker (Tmem8c)	F 5' CTGTGCTGTGCTTCATGC 3'
	R 5' GCCTATCTGCTGGGTGTAGA 3'
Murine Myomixer (Gm7325)	F 5' CTGAGCTCCCAAGACATGAG 3'
	R 5' CCAATCTCTCCTTCCTCTGG 3'
Murine ID3	F 5' ATGAAGGCGCTGAGCCCG 3'
	R 5' GTGGCAAAAGCTCCTCTTGTC 3'
Murine NP1	F 5' TTCTCCTCTCCCCTGCGG 3'
	R 5' GCACGTAAGTCCACTGCG 3'
Murine Tnnt1	F 5' GGAGAAGATGCGGAAGGAG 3'
	R 5' CAGTCTGTCGCTTCCCAC 3'

Chromatin immunoprecipitation (ChIP) primers:

Murine Igh	F 5' GCCGATCAGAACCAGAACACCTGC 3'
	R 5' TGGTGGGGCTGGACAGAGTGTTTC 3'
Murine Lmod2 Promoter	F 5' CACCCTCTCCACATTGTCAC 3'
	R 5' AAAGAACCAGGCATTCAAGG 3'
Murine Myh3 Promoter	F 5' CCTTTCTCTTCAGGCCACTAC 3'
	R 5' TGACAGGGAGCTATGCCA 3'
Murine Tnni2 Promoter	F 5' GCCAAAGGAGCAAGAGTTAAAAAT 3'
	R 5' AGGAGAAAGTGTTCCCAAAATGTC 3'
