# Supplementary figures and images for: Myogenin is required for assembly of the transcription machinery on muscle genes during skeletal muscle differentiation
Source: PLoS One. 2021 Jan 19;16(1):e0245618. doi: 10.1371/journal.pone.0245618 (PMC7815108; doi:10.1371/journal.pone.0245618)

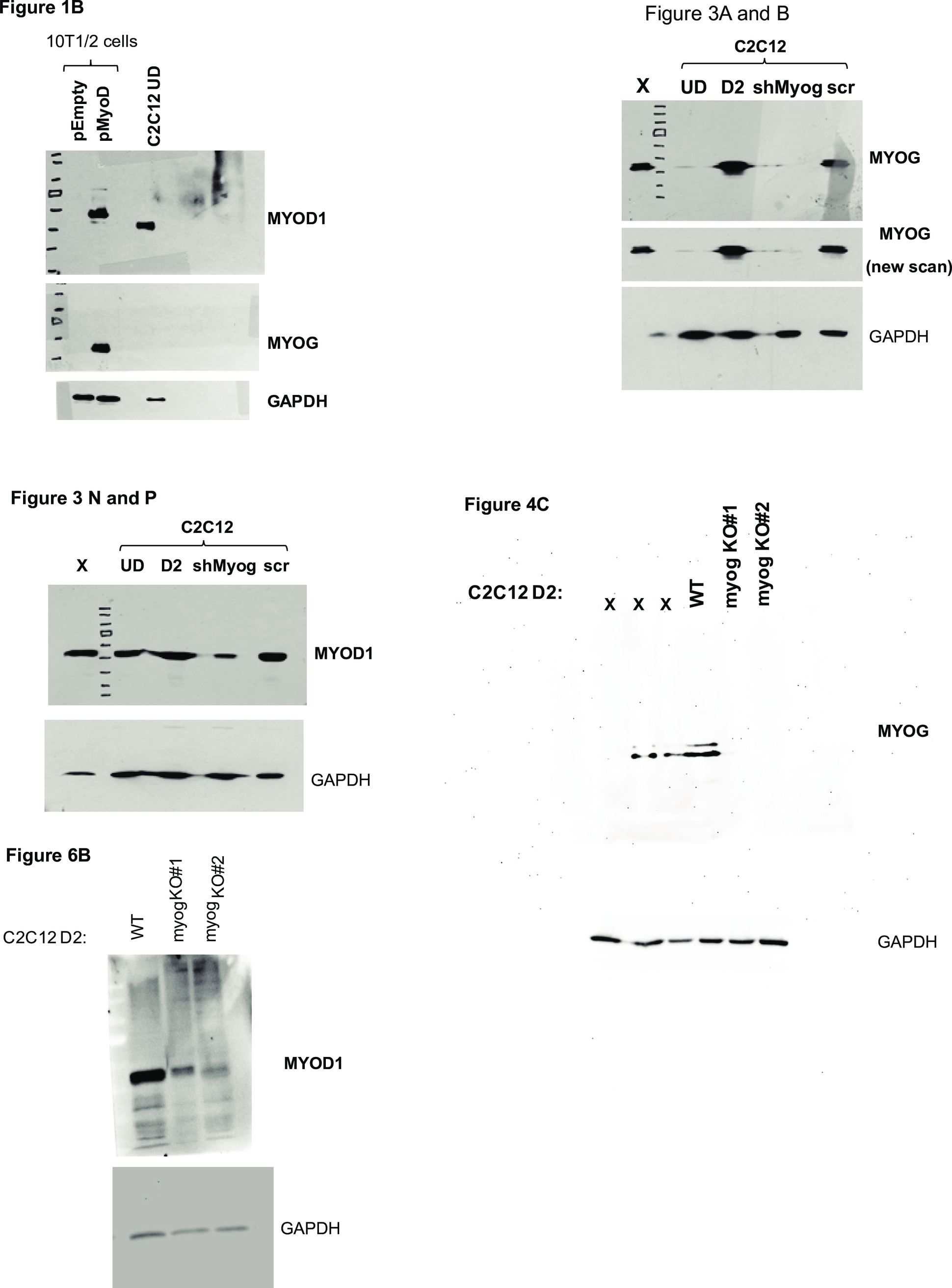

Supplement: S1 Raw images — (TIF) [file pone.0245618.s002.tif]
